# Isohydricity of Two Different Citrus Species under Deficit Irrigation and Reclaimed Water Conditions

**DOI:** 10.3390/plants10102121

**Published:** 2021-10-06

**Authors:** Cristina Romero-Trigueros, Jose María Bayona Gambín, Pedro Antonio Nortes Tortosa, Juan José Alarcón Cabañero, Emilio Nicolás Nicolás

**Affiliations:** Irrigation Department, Centro de Edafología y Biología Aplicada del Segura, CSIC, P.O. Box 164, 30100 Murcia, Spain; jmbayona@cebas.csic.es (J.M.B.G.); panortes@cebas.csic.es (P.A.N.T.); jalarcon@cebas.csic.es (J.J.A.C.); emilio@cebas.csic.es (E.N.N.)

**Keywords:** ABA, grapefruit, hydraulic conductance, mandarin, saline stress, stomatal conductance, water relations, water stress

## Abstract

Citrus species are frequently subjected to water and saline stresses worldwide. We evaluated the effects of diurnal changes in the evaporative demands and soil water contents on the plant physiology of grapefruit and mandarin crops under saline reclaimed (RW) and transfer (TW) water conditions, combined with two irrigation strategies, fully irrigated (fI) and non-irrigated (nI). The physiological responses were different depending on the species. Grapefruit showed an isohydric pattern, which restricted the use of the leaf water potential (Ψ_l_) as a plant water status indicator. Its water status was affected by salinity (RW) and water stress (nI), mainly as the combination of both stresses (RW-nI); however, mandarin turned out to be relatively more tolerant to salinity and more sensitive to water stress, mainly because of its low hydraulic conductance (K) levels, showing a critical drop in Ψ_l_ that led to severe losses of root–stem (K_root–stem_) and canopy (K_canopy_) hydraulic conductance in TW-nI. This behavior was not observed in RW-nI because a reduction in canopy volume as an adaptive characteristic was observed; thus, mandarin exhibited more anisohydric behavior compared to grapefruit, but isohydrodynamic since its hydrodynamic water potential gradient from roots to shoots (ΔΨ_plant_) was relatively constant across variations in stomatal conductance (g_s_) and soil water potential. The g_s_ was considered a good plant water status indicator for irrigation scheduling purposes in both species, and its responses to diurnal VPD rise and soil drought were strongly correlated with K_root–stem_. ABA did not show any effect on stomatal regulation, highlighting the fundamental role of plant hydraulics in driving stomatal closure.

## 1. Introduction

Citrus species are some of the most important commercial fruit crops around the world, including in semi-arid Mediterranean regions [1], where the irrigation water is not always available due to water scarcity; therefore, many citrus orchards suffer severe drought periods [2]. In order to overcome this issue, the use of non-conventional water sources such as reclaimed water (RW), whose volume is progressively increasing, is an alternative for farmers [3]. Among the advantages of the agronomic use of RW is the availability of macronutrients (N, P, K) as fertilizer, although there is a risk that these nutrients, for instance NO_3_, could be lost in the ecosystem through leaching [4]. The use of RW may, nevertheless, have risks for agriculture, since it could be highly saline; thus, inappropriate management of irrigation with RW can exacerbate problems of secondary salinization and soil degradation over the medium to long term, resulting in negative impacts on plant physiology, growth, and other factors [5,6,7]. Studies have shown that citrus plants in general are strongly affected by both drought and salinity, since Cl^-^ and Na^+^ can be phytotoxic [5,8]. The physiological effects of these stresses in citrus include, among others, a reduction of gas exchange [9] caused by a combination of factors. On the one hand, the availability of soil water and atmospheric vapor pressure are among the numerous environmental factors affecting the stomatal aperture in dry conditions (water-stressed citrus) [10]. The sensitivity to atmospheric vapor pressure deficit (VPD) is a primary strategy by which plants regulate gas exchange [11]. An increase in VPD or a reduction in soil water content leads to a decrease in stomatal conductance (g_s_) or a hydraulic cascade of water potential in the tree, which becomes larger and longer-lasting when high atmospheric water demand combines with soil water stress [12]. This ability of plants to regulate transpirational water loss and to minimize fluctuations in water potential defines plants as isohydric or anisohydric [13]. More isohydric species are prone to carbon starvation, while more anisohydric species are more likely to die from tissue desiccation via hydraulic failure [14]. Notwithstanding, the precise physical mechanism by which g_s_ and water potential are coordinated remains a matter of debate [15,16]. The isohydric–anisohydric behavior and its implications in the photosynthesis of different vine cultivars [17] and in the kinetics of stomatal opening of other woody species [18] have been studied; however, we have not found published studies that have defined this phenomenon in mandarin or grapefruit trees, although in general citrus plants are considered isohydric [19]. On the other hand, in salt-stressed citrus trees, decreased photosynthetic rates could be also associated with salt-induced reductions in CO_2_ diffusion to stomata, as was reported earlier [20]. It has been proven that being exposed to salt may affect plant metabolism through (i) a specific ion effect, causing a gradual accumulation of toxic Cl^−^ and Na^+^ levels in aerial parts when no compartmentation of ions in the vacuole takes place, or through (ii) an osmotic effect, causing a water deficit. One of the main mechanisms that plants use to adapt to osmotic stress is osmotic adjustment (OA), which can maintain the positive leaf turgor, also named as pressure potential (Ψ_P_), required to keep stomata open and sustain gas exchange [21], since stomatal closure could be affected by changes in the dynamic guard cells Ψ_P_ via feedback regulation [22]. Moreover, the role of hormone abscisic acid (ABA) in regulating g_s_ is also an enduring controversy [23,24]. Although so far ABA appears to be the main factor involved in regulation of stomatal closure under water stress [25,26,27], there is considerable evidence that plants are able to respond directly to hydraulic signals caused by water deficit [28,29]. The hydraulic signals may be involved in responses of stomata to decrease water potential [30,31], as cited above, or in the reduction of hydraulic conductance (K) during stress [32]. It has been demonstrated that seasonal shifts in K contribute to changes in g_s_ [33]; however, it is unclear whether naturally occurring diurnal changes in K influence stomata [34].

Some of the responses described so far do not always occur simultaneously in citrus species, as some are rootstock dependent [35,36,37]; thus, the tolerance or sensitivity to drought and salinity is determined by the rootstock [38,39], while its importance under the interaction of both stresses has not been reported yet to our knowledge. Further, studies that have evaluated tolerances in woody crops after extended periods of time applying these stresses are few in number because of the cost and time required. Consequently, understanding how saline and water stresses affect the dynamics of plant–atmosphere vapor exchange through its diurnal effects on g_s_ [40] is important within the context of climate change increasing drought [41] and VPD [11] occurrence worldwide. 

This study aims to evaluate the effects of long-term irrigation with saline RW and of the total suppression of irrigation for a period, as well as the combination of both stresses (saline and water), on diurnal changes in the physiology (water relations, phytotoxic elements, hydraulic conductance, ABA) of two citrus species with different rootstocks and productive potential, namely grapefruit [42] and mandarin [43], under field conditions. Additionally, we assess the stomatal response to environmental and physiological factors with the aim of finding useful indicators of plant water status for irrigation scheduling purposes. A fundamental aspect is to characterize the near isohydric or anisohydric behavior of both species. We hypothesize that the most physiologically affected treatment will be the one that combines both stresses, and in the case that both citrus species are isohydric, g_s_ would be a better indicator than Ψ_l_.

## 2. Results

### 2.1. Effects of the Irrigation Strategies on Water Relations, Hydraulic Conductance, and ABA

**Figure 1:**
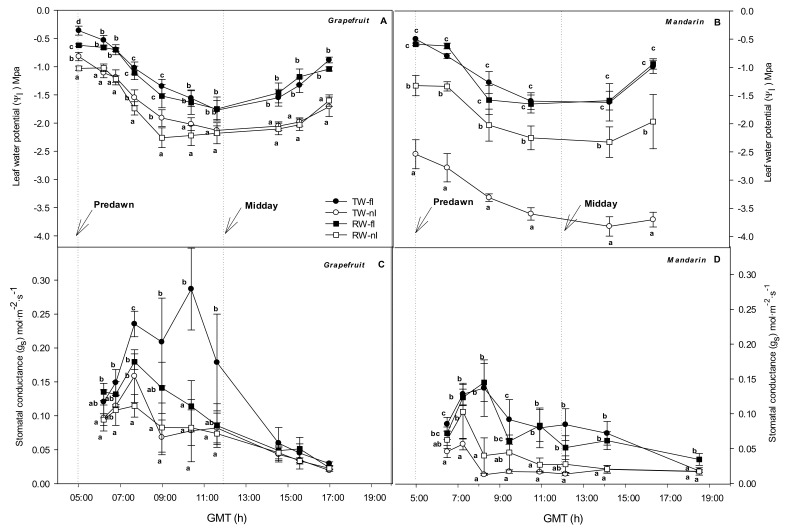
Daily evolution of leaf water potential (Ψ_l_) (**A**,**B**) and stomatal conductance (g_s_) (**C**,**D**) for each treatment (TW-fI: transfer water—fully irrigated; TW-nI: transfer water—non-irrigated; RW-fI: reclaimed water—fully irrigated; RW-nI: reclaimed water—non-irrigated) and crop (grapefruit and mandarin). Each point is the average ± standard deviation of 4 blocks, collected at 248 DOY. Different letters indicate significant differences at *p* < 0.05, as assessed using Tukey’s test.

The fully irrigated (fI) treatments of the grapefruit and mandarin crops resulted in similar Ψ_l_ values throughout the day regardless of the water quality, although in grapefruit crops the RW-fI values were slightly lower than for control (TW-fI). The non-irrigated (nI) treatments for both crops resulted in lower Ψ_l_ values than control at each sampling point. The most stressed treatments were RW-nI in grapefruit and TW-nI in mandarin, with the latter reaching values of about −3.8 MPa (Figure 1A,B). The Ψ_l_ measured at predawn (henceforth Ψ_soil_) was reduced in the nI treatments of both crops and also in RW-fI of grapefruit.

Regarding gas exchange (Figure 1C,D), in grapefruit, control trees reached the daily maximal value of g_s_ (0.287 mol·m^−2^·s^−1^) at midday (10.30 GMT); however, for the rest of stressed treatments, the maximal values were observed earlier (07.40 GMT) and were significantly lower than those of control (g_s_ reduced by 44.7, 37.4, and 60.0% for TW-nI, RW-fI, and RW-nI, respectively). In mandarin, control and RW-fI trees reached the daily maximal values of g_s_ (0.141 mol·m^−2^·s^−1^) at around 08.15 GMT; however, similarly to grapefruit, the nI trees reached the daily maximal values of g_s_ earlier (07.15 GMT), which were significantly lower than those of control (g_s_ reduced by 58.8 and 24.8% for TW-nI and RW-nI, respectively). Similar behaviors for both crops were observed over the rest of sampling that was carried out during the two months of irrigation suppression (Supplementary Figure S1). Overall, the treatments that were more affected by the irrigation strategies and daily VPD changes were: RW-nI in the grapefruit trees, as we hypothesized; TW-nI in mandarin trees (Figure 1, Figures S1 and S2). Grapefruit trees showed higher rates of g_s_ than the mandarin trees.

**Figure 2:**
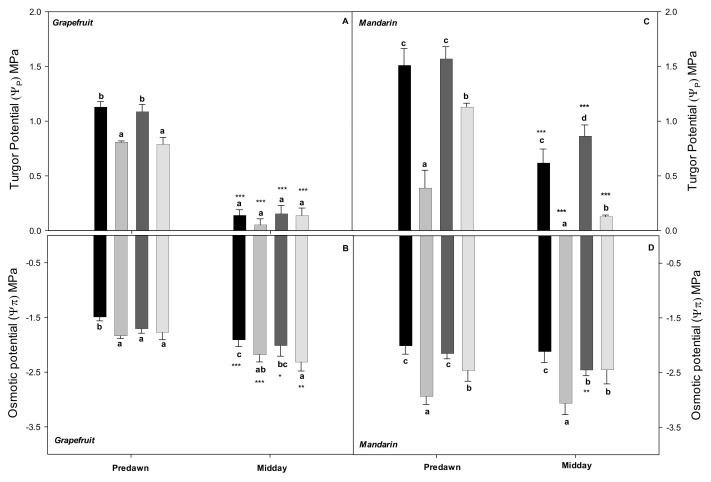
Leaf turgor potential (Ψ_P_) (**A**,**C**) and leaf osmotic potential (Ψ_π_) (**B**,**D**) at predawn and midday for each treatment (TW-fI: transfer water—fully irrigated; TW-nI: transfer water—non-irrigated; RW-fI: reclaimed water—fully irrigated; RW-nI: reclaimed water—non-irrigated) and crop (grapefruit and mandarin). Each value is the average of 4 blocks, collected at 248 DOY. The bars denote the standard deviation of the mean. Within each sampling and crop, different letters indicate significant differences at *p* < 0.05, as assessed using Tukey’s test. Asterisks indicate significant differences between time samplings for the same treatment according to repeated measures ANOVA (*** *p* < 0.001, ** *p* < 0.01, * *p* < 0.05).

Concerning the effects of the irrigation strategies on the Ψ_l_ components (Figure 2), in grapefruit trees, the Ψ_π_ values from all stressed treatments significantly decreased with respect to control at predawn (before the climatic conditions affected the plant water status) and at midday (except RW-fI). The treatment with the most negative Ψ_π_ values was RW-nI. The Ψ_P_ values were similar in the fI treatments (RW-fI and TW-fI) and significantly lower in the nI treatments at predawn. No significant differences among any treatments were shown at midday, since the Ψ_P_ values of the fI treatments significantly decreased (Figure 2A,B). In the mandarin crop, the Ψ_π_ values of fI treatments (TW-fI and RW-fI) were similar at predawn according to Ψ_soil_, although at midday the RW-fI values significantly decreased. The Ψ_π_ values of the nI treatments were significantly lower than those of control at both sampling points, with the TW-nI treatment being the most affected. The Ψ_P_ values of RW-fI were significantly higher than the control at midday. On the contrary, the nI treatments showed significantly decreased Ψ_P_ values, with TW-nI specifically showing the lowest Ψ_P_ values, which were near zero at midday (Figure 2C,D). In general, the mandarin trees presented lower Ψ_π_ and higher Ψ_P_ values than the grapefruit trees.

According to Ψ_100 s_ (Table 1), a moderate OA resulted from the RW-nI treatment (0.27 MPa) of grapefruit trees and from both nI treatments (0.25 and 0.15 MPa for TW-nI and RW-nI, respectively) of mandarin. Regarding phytotoxic elements (Table 1), in grapefruit there were no significant differences in the Cl^−^ and Na^+^ contents among treatments, although the values tended to be higher in the RW trees with respect to the control (by 29% and 32% for Cl^−^ and Na^+^, respectively, in RW-fI; by 48% for Na^+^ in RW-nI). In mandarin, the RW trees significantly increased the Cl^−^ (by 255% and 205% for RW-fI and RW-nI, respectively) and Na^+^ (by 52 and 59% for RW-fI and RW-nI, respectively) values versus the control, although only the Cl^−^ changes were significant.

Plant hydraulic conductance was significantly affected by water amount. In grapefruit, there was a significant decrease in the K_root–stem_ of the both nI treatments (TW-nI and RW-nI) and in the K_canopy_ of RW-fI, with respect to the control (Table 2). In mandarin, there was an important decrease in the K_root–stem_ of both nI treatments (TW-nI and RW-nI) and in the K_canopy_ of TW-nI versus control. 

Across treatments, in grapefruit trees the K_canopy_ was higher than the K_root–stem_ (the average values were 7.8 and 3.5 mol·MPa^−1^·m^−2^·s^−1^ for the K_canopy_ and K_root–stem_, respectively). On the contrary, in mandarin trees the average K_root–stem_ values were slightly higher than the K_canopy_ values (2.9 and 2.2 mol·MPa^−1^·m^−2^·s^−1^ for the K_root–stem_ and K_canopy_, respectively). Overall, the hydraulic conductance of mandarin crop was lower than that of grapefruitcrop, with mandarin trees also being more affected by water stress than grapefruit trees. In grapefruit, the k_root–stem_ values were reduced by 35.9 and 50.5% for TW-nI and RW-nI, respectively. In mandarin, the k_root–stem_ and k_canopy_ values were reduced by 85.2 and 38.7% for TW-nI and by 82.3 and 2.4% for RW-nI, respectively, versus control).

**Figure 3:**
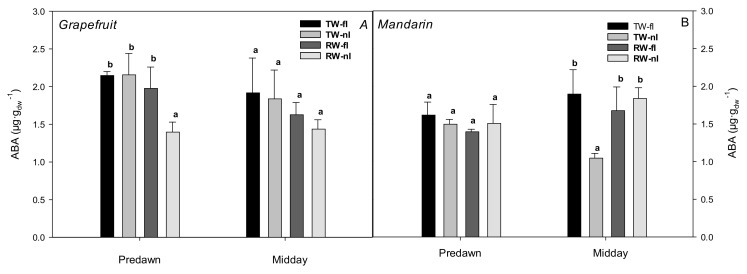
Leaf ABA content for grapefruit (**A**) and mandarin (**B**) and for each treatment (TW-fI: transfer water—fully irrigated; TW-nI: transfer water—non-irrigated; RW-fI: reclaimed water—fully irrigated; RW-nI: reclaimed water—non-irrigated). Each value is the average of 4 blocks, collected at 248 DOY. The bars denote the standard deviations of the mean. Within each sampling and crop, different letters indicate significant differences at *p* < 0.05, as assessed using Tukey’s test.

Regarding the phytohormonal signals of grapefruit trees, RW-nI resulted in lower ABA values than the rest of the treatments at both sampling points, although they were only significant at predawn. In mandarin trees, the ABA content was significantly lower for TW-nI at midday (Figure 3A,B); thus, decreased ABA levels were observed for the most water-stressed treatments (RW-nI in grapefruit and TW-nI in mandarin, Figure 1A,B). 

### 2.2. Relationship between Stomata and Environmental and Plant Physiological Factors

Multiple variables that can influence the stomatal response have been studied, which are classified here as environmental (soil water content and VPD) and physiological (non-hydraulic or hydraulic) factors. The group of non-hydraulic variables includes chemical signals such as the ABA, while the group of hydraulic variables includes Ψ_l_, T_l_, Ψ_π_, Ψ_P_, and K.

**Figure 4:**
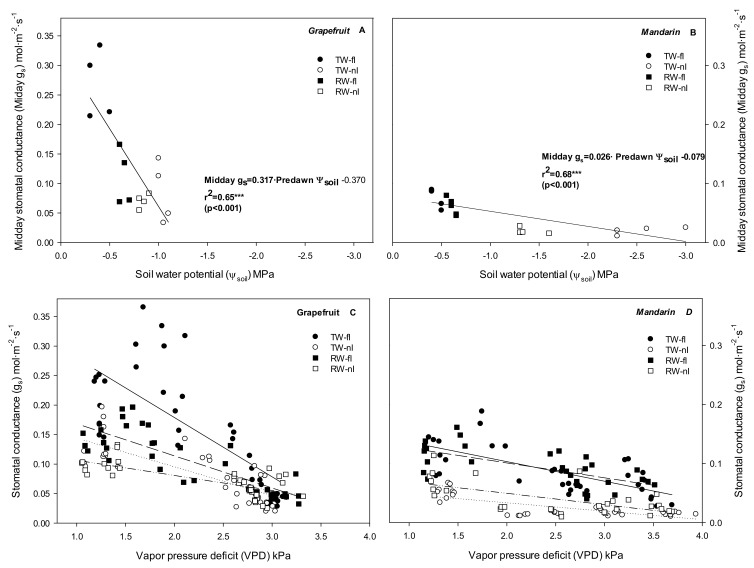
Correlations between midday stomatal conductance (Midday g_s_) and soil water potential (Ψ_soil_) values (**A**,**B**) and between stomatal conductance (g_s_) and vapor pressure deficit (VPD) values (**C**,**D**) for each treatment (TW-fI: transfer water—fully irrigated; TW-nI: transfer water—non-irrigated; RW-fI: reclaimed water—fully irrigated; RW-nI: reclaimed water—non-irrigated) and crop (grapefruit and mandarin). Each point is the average of the two central trees of each block. The regression lines between g_s_ and VPD for grapefruit plants were (**C**): TW-fI: g_s_ = −0.100·VPD + 0.170; r^2^ = 0.53 *** (*p* < 0.001); TW-nI: g_s_ = −0.049·VPD + 0.223; r^2^ = 0.61 *** (*p* < 0.001); RW-fI: g_s_ = −0.054·VPD + 0.193; r^2^ = 0.69 *** (*p* < 0.001); RW-nI: g_s_ = −0.026·VPD + 0.132; r^2^ = 0.52 *** (*p* < 0.001). The regression lines between g_s_ and VPD for mandarin plants were (**D**): TW-fI: g_s_ = −0.033·VPD + 0.170; r^2^ = 0.48 *** (*p* < 0.001); TW-nI: g_s_ = −0.014·VPD + 0.062; r^2^ = 0.55 *** (*p* < 0.001); RW-fI: g_s_ = −0.025·VPD + 0.152; r^2^ = 0.42 *** (*p* < 0.001); RW-nI: g_s_ = −0.019·VPD + 0.088; r^2^ = 0.39 *** (*p* < 0.001).

Regarding environmental factors, we found that the g_s_ was significantly correlated with the Ψ_soil_ (*p* < 0.001) (Figure 4A,B) and VPD (*p* < 0.001) (Figure 4C,D) in both crops. The linear regression plot for mandarin trees showed a gentler slope than that of grapefruit trees due to the Ψ_soil_ reaching very negative values and to the low g_s_ of mandarin versus grapefruit trees. The regression lines between g_s_ and VPD also showed different slopes according to treatment. In grapefruit trees, the slope was significantly steeper in the control than in the rest of treatments (TW-fI **>** RW-fI > TW-nI > RW-nI). Likewise, the slopes for mandarin plants were steeper in the fI treatments than in nI treatments (TW-fI > RW-fI > RW-nI > TW-nI), with RW-fI and TW-fI being quite similar (0.033 and 0.025); therefore, in both crops, the stomatal closure was more sensitive to VPD variations, mainly when soil water was not a very limiting factor. It is noteworthy that when plotting g_s_ and VPD data measured during the whole growth season between 06.00 and 08.00 GMT, no significant correlations between the parameters were found in any crop.

**Figure 5:**
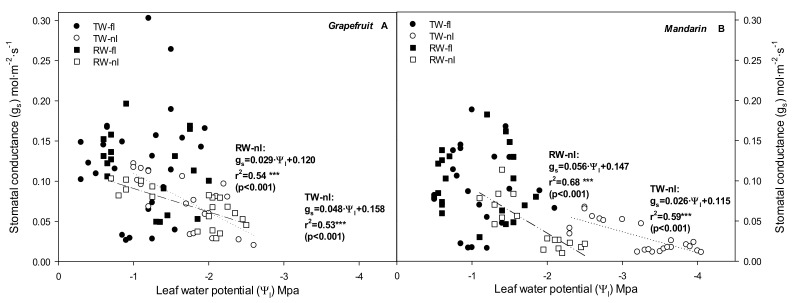
Correlations between stomatal conductance (g_s_) and leaf water potential (Ψ_l_) values for grapefruit (**A**) and mandarin (**B**) and all treatments. Each point is the average of the two central trees of each block.

Regarding physiological plant factors, the ABA results (non-hydraulic factor) did not correlate with g_s_ for any sampling, treatment, or crop. With respect to the physiological hydraulic factors, when soil moisture was not a limiting factor, regardless of water quality, the Ψ_l_ did not exert much control over g_s_ with the diurnal increase in VPD. In contrast, the nI treatments of both crops presented sensitive g_s_ responses to changes in Ψ_l_ throughout the day (Figure 5A,B).

**Figure 6:**
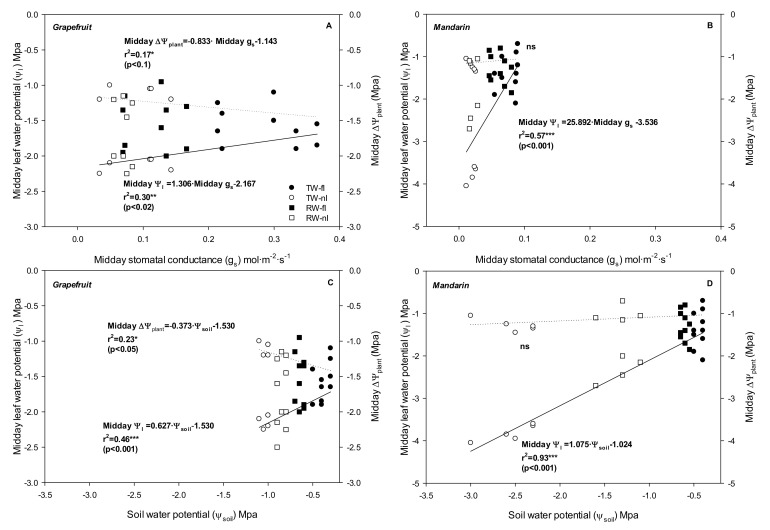
Correlations of midday leaf water potential (midday Ψ_l_) with midday stomatal conductance (midday g_s_) (continuous lines), of midday hydrodynamic (transpiration-induced) water potential gradient from roots to shoots (midday ΔΨ_plant_) with midday g_s_ (dash lines) (**A**,**B**), and of midday Ψ_l_ with soil water potential (Ψ_soil_) (continuous lines) and midday ΔΨ_plant_ with Ψ_soil_ (dash lines) (**C**,**D**) for all treatments and both crops (grapefruit and mandarin).

When only midday data were used, both crops showed positive significant correlations between midday Ψ_l_ and midday g_s_ (*p <* 0.02 for grapefruit and *p* < 0.001 for mandarin) (Figure 6A,B) and between midday Ψ_l_ and Ψ_soil_ (*p <* 0.001 both crops) (Figure 6C,D); however, the hydrodynamic (transpiration-induced) water potential gradient from roots to shoots (∆Ψ_plant_) was relatively constant in the mandarin crop. In fact, linear regression of midday ΔΨ_plant_ versus midday g_s_ or even Ψ_soil_ produced completely horizontal lines in mandarin trees (Figure 6B,D). This behavior was not observed in grapefruit trees (Figure 6A,C).

A positive relationship between g_s_ and T_l_ was found when only data below 30 °C were plotted (r^2^ = 0.51, *p <* 0.005 and r^2^ = 0.27, *p <* 0.005 for grapefruit and mandarin, respectively). This was due to the fact that from 20 to 30 °C, the VPD and T_l_ were linearly correlated (Figure S3), while from 30 to 40 °C, the VPD increased quickly and the g_s_ was widely dispersed, depending on the treatment. In general, the VPD and T_l_ values were greater in mandarin than in grapefruit trees.

**Figure 7:**
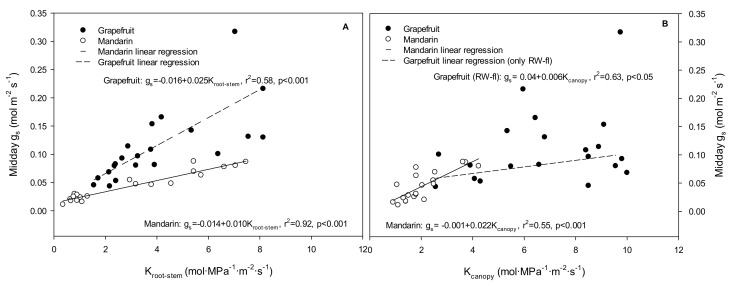
Correlations between midday stomatal conductance (midday g_s_) and (**A**) root–stem hydraulic conductance (K_root–stem_) and (**B**) canopy hydraulic conductance (K_canopy_) for each treatment (TW-fI: transfer water—fully irrigated; TW-nI: transfer water—non-irrigated; RW-fI: reclaimed water—fully irrigated; RW-nI: reclaimed water—non-irrigated) and crop (grapefruit and mandarin).

Finally, the stomatal response to the hydraulic conductance at midday was also studied (Figure 7). In grapefruit, the g_s_ was significantly correlated with the K_root–stem_ (r^2^ = 0.58, *p <* 0.001), while with the K_canopy_, this was true only for the RW-fI trees (r^2^ = 0.63, *p <* 0.05). In mandarin plants, g_s_ was correlated with both K_root–stem_ (r^2^ = 0.92, *p <* 0.001) and K_canopy_ (r^2^ = 0.55, *p <* 0.001). Across species, a significant correlation between g_s_ and K_root–stem_ (r^2^ = 0.50, *p <* 0.001) was found, but not for g_s_ and K_canopy_. 

## 3. Discussion

### 3.1. Water Relations of the Grapefruit and Mandarin Crops under Saline and Water Stresses

Mandarin and grapefruit presented different hydraulic conductance levels. Some studies reported K data for citrus plants with different rootstocks but using seedlings [36,44,45] or pots [46]. The higher hydraulic capacity, mainly of the canopy, observed in grapefruit compared to mandarin trees was explained by the high leaf gas exchange levels according to [47], who reported that species with large photosynthetic capacity must show a high hydraulic capacity to cope with the high g_s_ values required to avoid diffusional limitations to photosynthesis. Similar results were found by [48] for almond and olive plants, two species with different K values. Additionally, the K_root–stem_ values were more vulnerable to cavitation than the K_canopy_ values in both species, in agreement with [49], who showed that the root–stem segment is more prone to this process. The midday depression observed in g_s_ is common in many plant species [50]. It has been associated with variations in the midday stem water status [51], supporting the idea that the stomatal response to VPD is strongly related to the hydraulic characteristics of the whole plant, as well as the leaf [52]. Non-stomatal limitations such as decreased mesophyll conductance to CO_2_ may also be partly responsible for the midday depression, although it has been not demonstrated to predominate [53]. In our work, the higher the water deficit was, the lower the g_s_ values that were found and the earlier in the morning the maximum g_s_ was reached, in line with [54].

The strategies and resistance mechanisms developed by plants under the different irrigation strategies depended on the crop, which are described below.

In the grapefruit crop, the trees under water stress and previously irrigated with TW (TW-nI) showed a reduction of 0.5 MPa in Ψ_soil_. This resulted in a 35% drop in the K_root–stem_ value, decreasing the Ψ_l_ and Ψ_π_ as well as the leaf Ψ_P_ with respect to the control, in accordance with [6], thereby affecting the gas exchange. When trees were fully irrigated but with saline RW, the Ψ_soil_ was also reduced, although to a lesser extent (0.3 MPa). In this case, this was not due to water restrictions but to the soil salt accumulation from the RW source, which caused an osmotic effect in the root zone, hindering the absorption of water by the trees [55]; however, unlike TW-nI, this Ψ_soil_ drop caused cavitation of conductive elements of the canopy and the subsequent loss of the K_canopy_, in line with [56], which was the main cause of the decrease in g_s_ and was linked to Cl^−^ and Na^+^ toxicity. The g_s_ reduction largely prevented Ψ_l_ and Ψ_π,_ from being affected, according to other studies on citrus plants [7], allowing the leaf turgor to be maintained at similar levels to the control trees. The fact that Ψ_l_ did not decline measurably with g_s_ showed that stomata responded quickly and sensitively enough to hydraulic signals to achieve near-homeostasis in Ψ_l_ [34]. Consequently, Ψ_l_ was not a good indicator of actual plant water status for irrigation scheduling, differing from other fruit trees [57,58]. In the RW-nI treatment, the g_s_ reduction was a little more pronounced than for TW-nI, since the combination of both stresses caused greater decreases in the Ψ_soil_ (reduction of 0.7 MPa) and Ψ_l_ throughout the day, giving rise to an important loss of K_root–stem_ due to cavitation and embolism of xylem vessels [59]. Moreover, the *Citrus macrophylla* (grapefruit) rootstock lacked Cl^−^ and Na^+^ under salinity conditions, in accordance with [60] and with the salt tolerance rankings of the rootstocks reported by other authors [61,62]. Low Na^+^ and Cl^−^ levels in the grapefruit trees irrigated with saline RW as compared to the control did not affect the vegetative growth (similar canopy volumes in all trees as were described in [42] and [63]) or yield [7], suggesting that this crop is a salt-stress-tolerant citrus [64].

In the mandarin crop, the trees under irrigation suppression (TW-nI) showed drastic reductions in the Ψ_soil_ values (2 MPa less than control), showing severe water stress with very negative Ψ_l_ values achieved by means of decreases in Ψ_π_, according to [65]. This made it difficult to take up water from the substrate, affecting the K_root–stem_ and K_canopy_, and causing a strong reduction in Ψ_P_, (~0 MPa). This led to foliar folding, which is a mechanism of resistance that minimizes water loss [66], and later the point of wilting with defoliation symptoms. With these disorders, gas exchange was severely reduced. Under the suppression of irrigation, the Ψ_l_ values decreased much more in mandarin trees than in grapefruit trees (see Figure S2) due to mandarin plants generally having lower hydraulic conductance than grapefruit plants. Another study in two orange varieties found that the variety with reduced hydraulic conductivity presented more negative water potential and g_s_ values under the same high evaporative demand period [44]. As for mandarin trees fully irrigated with saline RW (RW-fI), contrary to what was observed in grapefruit trees, the gas exchange was not reduced by water quality because the Ψ_soil_, plant hydraulic conductance, and Ψ_l_ were unaffected. In spite of the Ψ_π_ decreasing slightly at some point, the high salinity in the leaves did not give rise to a specific ion effect, but could help to increase the leaf turgor versus control. Even though the Ψ_100 s_ data did not indicate an important OA in this treatment, when the Ψ_P_ of citrus plants under saline conditions is similar to or higher than that of control trees, Cl^−^ and Na^+^ accumulation represent OA processes, according to [67]. Finally, contrary to what we hypothesized, the nI treatment preconditioned by salinity stress (RW-nI) maintained a better water status during drought stress, since the Ψ_soil_, K, and Ψ_l_ were reduced much by much less than in TW-nI. This was justified because the canopy volume of RW-nI was less than that of TW-nI. The *Carrizo citrange* (mandarin) rootstock was a less effective Cl^−^ and Na^+^ excluder under salinity conditions [60]; that is, mandarin trees did not develop a strategy for the removal of saline ions from the RW source as grapefruit trees did, but instead opted for an osmotic strategy involving the accumulation of leaf Na^+^ and Cl^−^, affecting vegetative growth and yield [7]. Further, the RW-nI trees underwent acclimatization to salinity by reducing the drop in the water potential over several vegetative cycles for the same experimental plot, as reported in [68].

### 3.2. Relationship between Water Relations and Hydraulic Conductance: Near-Isohidric or Anisohydric Behavior

Citrus is considered isohydric in general, since it usually presents a stomatal sensitivity to water stress conditions [19]. In previous studies from the same experimental plots but with moderate deficit irrigation instead of total irrigation suppression [5,6,7,36,42,68], there was not a complete prioritization of the stomatal aperture for maintenance of CO_2_ assimilation. Additionally, the Ψ_s_ values did not vary widely throughout several growth seasons for the same treatment nor between treatments, indicating a possible typical isohydric behavior. Nevertheless, it should be considered that there may be more than one definition of isohydricity and that the different definitions are not always in agreement [69]. Consistent with the concept presented in [70], here both citrus species displayed a stomatal sensitivity to soil water and evaporative demands, although to different degrees. Grapefruit trees showed higher reference g_s_ (g_sRef_, corresponding to g_s_ at 1 kPa VPD) (g_sRef_ = 0.092 mol·m^−2^·s^−1^) than mandarin (g_sRef_ = 0.078 mol·m^−2^·s^−1^), suggesting that grapefruit trees tended to have a more sensitive response to increasing VPD (see slopes in Figure 4C,D). This lower stomatal sensitivity of mandarin to VPD and Ψ_soil_ could indicate a less isohydric behavior compared to grapefruit, according to the theory presented in [11]. Nonetheless, greater g_s_ regulation to prevent decreased water potential to levels that provoke excessive loss of hydraulic conductance was not possible due to the low gas exchange levels of mandarin trees, in accordance with [71]. The isohydric plant concept presented by [72] implies a similarity in midday Ψ_l_ values in nI and fI plants. This did not occur here in mandarin crop, in which the Ψ_l_ values varied by more than 1 MPa among treatments (Figure 1B), indicating again that the mandarin crop had a near anisohydric response as compared to the grapefruit crop. The apparent simplicity of the concept could lead to misinterpretation of isohydry as a simple functional trait or a strategy defined by the isolated action of the stomata and not a response of the entire plant in order to regulate the water status [69]. In our study, when the isohydricity concept was assessed within a whole-plant perspective, we found that the strong stomatal control maintained relatively constant internal water potential gradients (hydrodynamic showed by ∆Ψ_plant_) in mandarin trees, while at the same time allowing Ψ_l_ to fluctuate intensely on a diurnal basis in synchrony with Ψ_soil_. This pattern of hydraulic regulation of mandarin trees was defined as isohydrodynamic by [73]. According to our knowledge, this is the first time that these species have been defined in the literature. As grapefruit is isohydric and mandarin is more anisohydric than grapefruit but also isodydrodynamic, mandarin plants did not prioritize stomatal opening, and for this reason mandarin did not neatly fit into the anisohydric extremes. Frameworks that mathematically link isohydricity and sensitivity to VPD have recently been developed [74], but they await empirical validation of stomatal sensitivity to VPD in diurnal environmental gradients [11]. The difference observed here among both citrus crops are in agreement with decades of studies that have highlighted the fact that stomatal sensitivity to VPD is highly variable across species [75,76,77]. Generally, we found an important decrease in g_s_ with diurnally increasing VPD in all treatments, in agreement with other authors [78,79], although when the whole growth season was evaluated, the VPD had less influence on g_s_. Regarding the water stress, stomatal closure throughout the day was correlated with Ψ_l_ in addition to VPD in grapefruits plants by not completely avoiding a decrease in Ψ_l_, which also occurred to a lesser for mandarin plants. No consensus exists as to the exact sensing mechanisms driving the stomatal closure response to increased VPD [11]. Part of the uncertainty associated with the impacts of VPD on plants relates to the difficulty of disentangling the effects of VPD from those of T_l_ [80]. Additionally, few studies have documented the direct stomatal response to T_l_ [81]. Here, T_l_ had a positive effect on g_s_ only with T_l_ < 30 °C, in accordance with [11], who reported that when VPD is low and stomata are fully open, T_l_ increases linearly with VPD.

Moreover, our findings regarding K highlighted the fundamental role of plant hydraulics in driving stomatal closure [51] in response to high VPD at midday and high soil water variation; that is, the K_root–stem_ (in both crops) and K_canopy_ (in mandarin) values were strongly related to g_s_. So far, we have not found any studies on citrus trees with the rootstocks studied here, on irrigation with saline RW, or involving field trials. Nevertheless, other studies have reported results in the same direction as ours—reduced K at low water potential can enhance stomatal closure during drought [33], while at greater water stress levels, K_leaf_ [48] or K_plant_ [82] decreases with a concominant decline in g_s_ [56]. Regarding salinity, decreased K_root_ caused by NaCl in sour orange and Cleopatra mandarin [83] and reductions in K_root_ of seedlings of Cleopatra mandarin, Carrizo citrange, and Poncirus trifoliara under long-term salt treatments have been reported [84].

The loss of hydraulic plant functioning has been considered one of the main driving factors of stomatal closure recently [15]. In grapefruit, the fact that midday g_s_ was better-correlated with K_root–stem_ than with K_canopy_ suggested that g_s_ reductions under water stress (nI treatments) happened in response to certain root- or stem-based signals, in line with [49]. Additionally, the sensitivity of g_s_ to K_root–stem_ was 2.5 time higher in grapefruit than in mandarin, in which g_s_ responded more sensitively to K_canopy_. 

The other major mechanism considered to trigger stomatal closure is the increase in chemical signals such as ABA [85]. The water flow reduction through some parts of the roots, which might be hydraulically isolated due to severe water stress, could be related to a reduced release of root ABA into the leaf xylem [86]. This would explain why the lowest ABA values in our study were found with TW-nI treatment of mandarin; however, in recent experiments (although not in citrus) have shown strong stomatal responses to changes in water supply originating primarily in the leaves, but not in the roots [87,88]. Here, the fact that g_s_ did not correlate with leaf ABA suggested that the importance of ABA in controlling g_s_ had lower weight than hydraulic conductance. Other authors also found that the relationship of ABA with g_s_ was not significant under drought conditions in olive plants [48] and in almond, grapevine, and olive crops [33].

## 4. Materials and Methods

### 4.1. Experimental Conditions and Plant Materials 

The experiment was conducted on a commercial citrus orchard, located in the northeast of the Murcia region in Campotéjar, 7 km north of Molina de Segura (38°07′18″ N, 1°13′15″ W). There were two experimental plots measuring 0.5 ha each. The first was cultivated with adult 8 year-old Star Ruby grapefruit trees (Citrus paradisi Macf) grafted on Macrophylla (*Citrus macrophylla*) rootstock and planted at 6 × 4 m. The second plot contained adult 14-year-old mandarin trees (Citrus clementina cv. ‘Orogrande’) grafted on *Carrizo citrange* (Citrus sinensis (L.) Osb. × Poncirus trifoliata (L.)) rootstock and planted at 5 × 3.5 m. 

The irrigation was scheduled on the basis of daily evapotranspiration of the crop (ET_c_) accumulated during the previous week. ET_c_ values were estimated as reference evapotranspiration (ET_0_) values, calculated with the Penman–Monteith methodology and a monthly local crop factor [89]. All treatments included application of the same amounts of fertilizer (N–P_2_O_5_–K_2_O) applied through the drip irrigation system (see [90]). Weeds were eradicated in the orchard by applying the farmers’ commonly used pest control methods [63].

### 4.2. Water Sources and Irrigation Treatments

The experimental plot of grapefruit and mandarin plants was irrigated with two water sources of different quality over six years of cultivation. The first type of irrigation water was pumped from the Tajo–Segura canal (transfer water, TW), while the second water source came from the north of “Molina de Segura” tertiary wastewater treatment plant (WWTP) (reclaimed water, RW). The latter source was characterized by having high salt and electrical conductivity (EC) values close to 4 dS·m^−1^, while for TW the EC values were lower at close to 1 dS·m^−1^ (annual average value: 0.93 ± 0.14 dS·m^−1^). The saline water source was automatically blended in the irrigation control head with water from TW to reduce its EC value to approximately 3.5 dS·m^−1^ (annual average value: 3.73 ± 0.80 dS·m^−1^), as an intermediate value between the threshold for significant yield losses (1.5–2 dS·m^−1^) [91] and the average EC of 4 dS·m^−1^ at the outlet of the WWTP. This high level of salinity observed in RW was mainly due to the high concentrations of Cl^−^ (69.95 ± 22.04 and 639.31 ± 189.91 mg L^−1^ for TW and RW, respectively) and Na+ (46.03 ± 14.18 and 604.03 ± 121.65 mg L^−1^ for TW and RW, respectively) (see Table S1).

Two irrigation treatments were applied for each water source and crop. The first was the fully irrigated treatment (fI), with irrigation occurring throughout the growing season to fully satisfy the crops’ water requirements (100% of crop evapotranspiration, ET_c_). The second was the non-irrigated treatment (nI), with an irrigation regime similar to fI, except for a two month period during one growing season (from 214 to 274 DOY; that is, from 1^st^ August to 1^st^ October) in which trees were not irrigated (0% ET_c_). This period of total suppression of irrigation was selected, taking into account the months of high evaporative demands that allowed the plants to reach a state of high stress; therefore, four treatments (TW-fI, TW-nI, RW-fI, and RW-nI) were established for each crop (grapefruit and mandarin). TW-fI was considered as the control treatment in both crops. The annual amounts of water applied for grapefruit and mandarin trees were 5700 and 7940 m^3^·ha^−1^, respectively, for the fI treatments; and 4618 and 6375 m^3^·ha^−1^, respectively, for nI treatments.

### 4.3. Measurements

Physiological plant measurements were carried out periodically from 213 to 283 DOY (i.e., before the beginning of the total irrigation suppression period and until 9 days after the end) (Figure S1). When severe water stress was reached using some of the nI treatments, a diurnal evolution was performed (248 DOY, 34 days after the initiation of total irrigation suppression).

#### 4.3.1. Plant Water Status

Stomatal conductance (g_s_), leaf temperature (T_l_), vapor pressure deficit based on leaf temperature (VPD), stem water potential (Ψ_s_), leaf water potential (Ψ_l_), leaf osmotic potential (Ψ_π_), and leaf osmotic potential at full turgor (Ψ_100 s_) were determined on one mature leaf, which was fully expanded from the mid-shoot area of each tree. Here, Ψ_l_ was measured at predawn and was used as an estimate of soil water potential (Ψ_soil_).

The g_s_, T_l_, and VPD were measured with a portable photosynthesis system (LI-6400 Li-Cor, Lincoln, Nebraska, USA) equipped with a clear chamber bottom (6400-08) and a LICOR 6400-01 CO_2_ injector. The measurements were performed on leaves that were placed in a 6 cm^2^ leaf cuvette. The CO_2_ concentration in the cuvette was maintained at 400 µmol·mol^−1^ (≈ambient CO_2_ concentration). The measurements were carried out at ambient air temperature and relative humidity. Measurements were taken approximately every hour on the same leaf from 06.00 to 19.00 GMT.

The Ψ_s_ and Ψ_l_ were measured in a single mature leaf from the same region of the canopy using a Scholander-type pressure chamber (model 3000; Soil Moisture Equipment Corp.; Santa Barbara, California, US; [92]) and following the recommendations in [93]. The Ψ_s_ was measured at midday and the leaves were covered with aluminum foil and enclosed within polyethylene bags at least 2 h before collection and measurement [94]. The Ψ_l_ was measured periodically throughout the day. The leaves used to measure Ψ_l_ at predawn and at midday were frozen in liquid N (−196 °C) and stored at −30 °C. After thawing, Ψ_π_ was measured in the extracted sap using a WESCOR 5520 vapor pressure osmometer (Wescor Inc.; Logan, UT, US) according to [95]. The pressure potential (Ψ_P_) was calculated as the difference between Ψ_l_ and Ψ_π_. The leaf osmotic potential at full turgor (Ψ_100s_) was estimated at midday as indicated above for Ψ_π_, using excised leaves with their petioles placed in distilled water overnight to reach full saturation. The osmotic adjustment (OA) was calculated as the difference in Ψ_100 s_ between the control (TW-fI) and the rest of the treatments.

The canopy hydraulic conductance (K_canopy_) and root–stem conductance (K_root–stem_) were estimated using the evaporative flux method [96]. On the one hand, the K_canopy_ was calculated under steady-state conditions according to Ohm’s law,

K_canopy_ = T_r_ / Midday ΔΨ_stem-leaf_ (1)

where Midday ΔΨ_stem-leaf_ is the water potential drop (MPa) across the stem–leaf pathway, obtained as the difference between Ψ_s_ and Ψ_l_, both at midday. On the other hand, K_root–stem_ was also calculated according to Ohm’s law,

K_canopy_ = T_r_ / Midday ΔΨ_stem-leaf_ (2)

where Midday ΔΨ_root-stem_ is the water potential drop (MPa) across the root–stem pathway obtained as the difference between predawn Ψ_l_ and midday Ψ_s_. The hydrodynamic (transpiration-induced) water potential gradient from roots to shoots (∆Ψ_plant_) was calculated as the difference between predawn and midday Ψ_l_.

#### 4.3.2. Leaf Chemical Analysis

Leaf abscisic acid (ABA) and phytotoxic elements such as Na^+^ and Cl^−^ were determined on twenty mature leaves, which were fully expanded from the mid-shoot area in each tree. Leaf samples used to measure ABA concentrations were freeze-dried and finely ground. Deionized water was added at a 1:50 weight ratio. Samples extracts were analyzed using a radioimmunoassay [97] to obtain leaf ABA contents. The phytotoxic elements were determined as in [6].

### 4.4. Statistical Design and Analysis

The experimental design for each irrigation treatment involved 4 standard experimental plots distributed following a completely randomized design. Each replicate was made up of 12 trees, organized in 3 adjacent rows. A total of 192 grapefruit trees and 192 mandarin trees were used. All measurements were carried out in the two central trees of the middle row of each replicate (2 trees per block, 8 per treatment), while border trees were excluded from the study to eliminate potential edge effects.

The average values of each treatment were analyzed as assessed using Tukey’s test. The significance of determination coefficients (r^2^) from linear regression equations were indicated as Pearson correlation coefficients (R). The data were also analyzed with a two-way ANOVA for repeated measures to examine the interaction between the treatments and time samplings. Further, the data were analyzed using a two-way ANOVA with the quality and amount of water as the main factors. These statistical analyses were performed with IBM SPSS Statistics software (version 23.0 for Windows, SPSS Inc.; Chicago, IL, USA).

## 5. Conclusions

Water and saline stresses triggered physiological changes in citrus trees in response to increases in VPD and soil drought throughout the day. Nonetheless, the degree of affectation and the strategies were differently modulated depending on the particular stress tolerance of the citrus crop and rootstock. In grapefruit, the use of RW and the irrigation suppression negatively affected the plant physiology, with the treatment that combined both stresses (RW-nI) being the one that most affected water status. In mandarin, the trees under saline stress (RW-fI) accumulated salts, unlike grapefruit, as an osmotic strategy. Then, the leaf turgor improved and the gas exchange was maintained similarly to control. The treatment that most affected water status was not RW-nI, as we expected, but TW-nI. This treatment presented injury symptoms, similarly to senescence, which were the consequence of the depletion of soil water and a critical drop in the Ψ_l_ that led to a severe loss of K. Such behavior was not observed with RW-nI because a slight reduction in the canopy volume as an adaptive characteristic was found, suggesting that when mandarin trees under water stress were previously acclimated to saline stress they were more effective in avoiding harmful cavitation. Thus, from a physiological point of view (without taking into account fruit yield), mandarin trees tolerated drought less than grapefruit trees, mainly because of their low K levels. Additionally, the mandarin crop exhibited more anisohydric behavior compared with the grapefruit crop due to the noteworthy drop of Ψ_l_ under water stress. Nevertheless, from a whole-plant perspective (∆Ψ_plant_), a constant hydrodynamic pattern more typical of isohydric crops was found in mandarin, defining it as isohydrodynamic. Moreover, grapefruit showed an isohydric pattern that limited the use of Ψ_l_ as an indicator of plant water status. Here, the g_s_ did show as a good water status indicator for irrigation scheduling purposes and was negatively correlated with the hourly increase in VPD for both crops. Our ABA data added to a growing body of evidence challenging the ABA-centric model of stomatal responses to abiotic stress, with K being the key factor. Further studies focusing on leaf hydraulic conductivity are required to understand the role of hydraulics and its mechanism on stomatal regulation in citrus.

## Figures and Tables

**Table 1:** Leaf osmotic potential at full turgor (Ψ_100s_) at midday, osmotic adjustment (OA), and leaf phytotoxic element values for each treatment and crop (grapefruit and mandarin). Each value is the average ± standard deviation of 4 blocks collected at 248 DOY. Within each column, different letters indicate significant differences at *p* < 0.05, as assessed using Tukey´s test.

Crop	Treatment	Ψ_100s_ (MPa)	OA (MPa)	Cl^−^ (%)	Na^+^ (%)
Grapefruit	TW-fI	−1.59 ± 0.13 a	-	0.44 ± 0.02 a	0.050 ± 0.005 a
	TW-nI	−1.45 ± 0.11 a	−0.14	0.32 ± 0.11 a	0.036 ± 0.004 a
	RW-fI	−1.39 ± 0.21 a	−0.20	0.57 ± 0.02 a	0.066 ± 0.026 a
	RW-nI	−1.86 ± 0.01 b	0.27	0.40 ± 0.28 a	0.074 ± 0.038 a
Mandarin	TW-fI	−1.73 ± 0.11 a		0.18 ± 0.02 a	0.046 ± 0.010 a
	TW-nI	−1.98 ± 0.08 b	0.25	0.15 ± 0.07 a	0.055 ± 0.020 a
	RW-fI	−1.69 ± 0.10 a	0.04	0.64 ± 0.08 b	0.070 ± 0.009 a
	RW-nI	−1.88 ± 0.03 ab	0.15	0.55 ± 0.05 b	0.073 ± 0.006 a

TW-fI: transfer water—fully irrigated; TW-nI: transfer water—non-irrigated; RW-fI: reclaimed water—fully irrigated; RW-nI: reclaimed water—non-irrigated.

**Table 2:** Root–stem hydraulic conductance (K_root–stem_) and canopy hydraulic conductance (K_canopy_) values for each treatment and crop (grapefruit and mandarin). Each value is the average ± standard deviation of 4 blocks, collected at 248 DOY. Within each column, different letters indicate significant differences at *p* < 0.05, as assessed using Tukey’s test. In two-way ANOVA, including water quality (Qw) and amount (Aw) as factors, *** *p* < 0.001, ** *p* < 0.01, * *p* < 0.05; ns: not significant.

Crop	Treatment	K_root–stem_ (mol·MPa^−1^·m^−2^·s^−1^)	K_canopy_ (mol·MPa^−1^·m^−2^·s^−1^)
Grapefruit	TW-fI	4.51 ± 0.28 b	8.93 ± 1.81 b
	TW-nI	2.89 ± 0.29 a	8.47 ± 2.26 b
	RW-fI	4.54 ± 0.22 b	4.26 ± 0.93 a
	RW-nI	2.23 ± 0.20 a	9.65 ± 3.20 b
ANOVA	Qw	ns	ns
	Aw	*	*
	Qw * Aw	ns	*
Mandarin	TW-fI	4.87 ± 0.81 b	2.47 ± 0.81 b
	TW-nI	0.69 ± 0.11 a	1.51 ± 0.23 a
	RW-fI	5.30 ± 0.38 b	2.48 ± 0.43 b
	RW-nI	0.86 ± 0.18 a	2.41 ± 1.26 b
ANOVA	Qw	ns	ns
	Aw	***	**
	Qw * Aw	ns	ns

TW-fI: Transfer water—fully irrigated; TW-nI: Transfer water—non-irrigated; RW-fI: Reclaimed water—fully irrigated; RW-nI: Reclaimed water—non-irrigated.

## Data Availability

Data is contained within the article and Supplementary Materials.

## Supplementary Materials

The following are available online at www.mdpi.com/2223-7747/10/10/2121/s1, Figure S1: Annual data for rainfall, reference evapotranspiration (ET_0_), and vapor pressure deficit based on the air temperature (VPD_air_); Figure S2: Relation between leaf water potential (Ψ_l_) and vapor pressure deficit (VPD) for each treatment; Figure S3. Relationship between vapor pressure deficit (VPD) and leaf temperature (T_l_) for each crop (grapefruit and mandarin); Table S1: Chemical parameters of each irrigation water source: Transfer Water (TW) and saline Reclaimed Water (RW).
