# Supplementary material for: Isohydricity of Two Different Citrus Species under Deficit Irrigation and Reclaimed Water Conditions
Source: Plants (Basel). 2021 Oct 6;10(10):2121. doi: 10.3390/plants10102121 (PMC8538605; doi:10.3390/plants10102121)
Supplement: Supplementary file 1 [file plants-10-02121-s001.zip › plants-1375113-supplementary.pdf]

Supplementary Materials

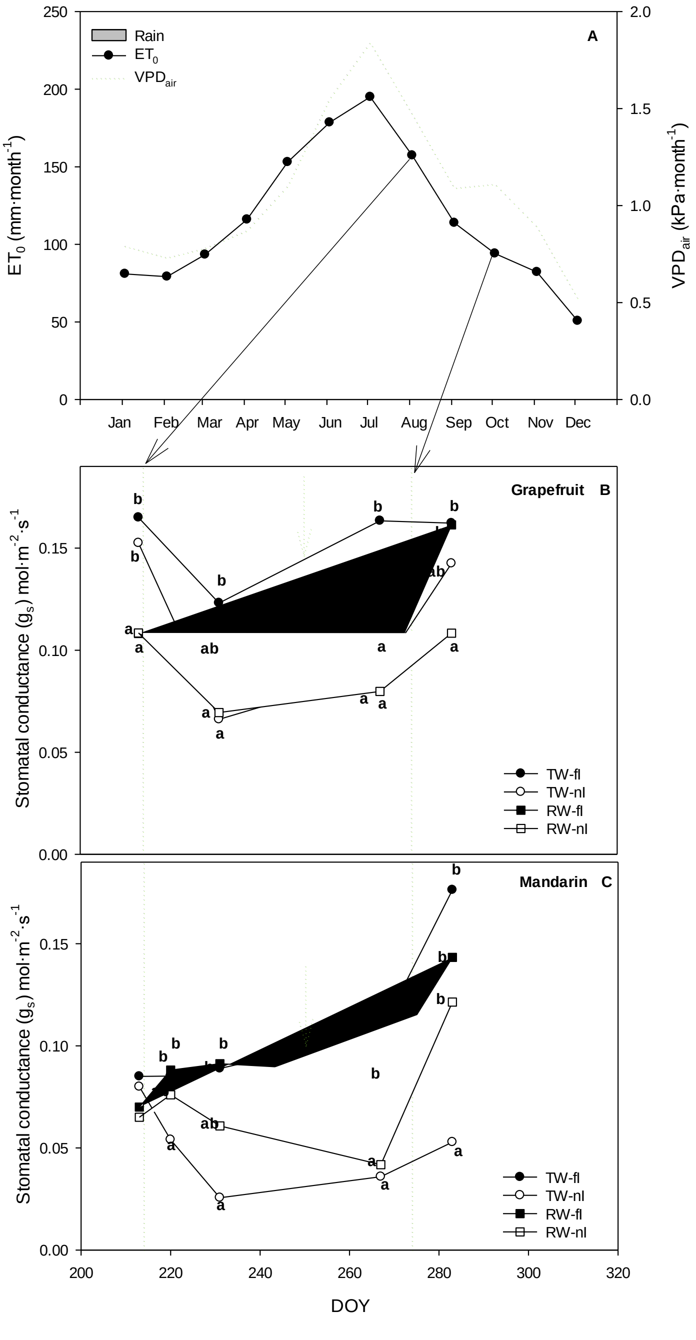

Figure S1. Annual data for rainfall, reference evapotranspiration (ET_0), and vapor pressure deficit based on the air temperature (VPD_{air}) (**A**) and stomatal conductance (g_s) evolution for each treatment (TW-fI: transfer water—fully irrigated; TW-nI: transfer water—non-irrigated; RW-fI: reclaimed water—fully irrigated; RW-nI: reclaimed water—non-irrigated) and crop, namely grapefruit (**B**) and mandarin (**C**). Each point is the average of 4 blocks. Different letters indicate significant differences at $p < 0.05$, as assessed using Tukey's test. The dotted arrow indicates the day of the diurnal evolution (DOY 248).

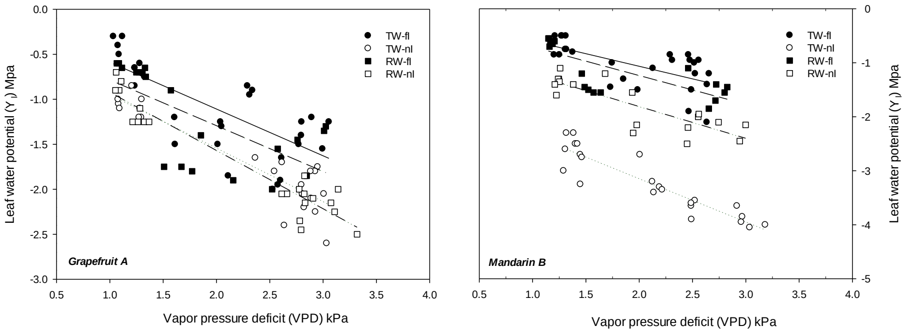

Figure S2. Relation between leaf water potential (Ψ_l) and vapor pressure deficit (VPD) for each treatment (TW-fI: transfer water—fully irrigated; TW-nI: transfer water—non-irrigated; RW-fI: reclaimed water-full-Irrigated; RW-nI: reclaimed water—non-irrigated) and crop (grapefruit and mandarin). Regression lines for grapefruit were: TW-fI: $\Psi_l = -0.520$ VPD -0.069; $r^2 = 0.55$ *** ($p < 0.001$); TW-nI: $\Psi_l = -0.594 \cdot$ VPD -0.352; $r^2 = 0.84$ *** ($p < 0.001$); RW-fI: $\Psi_l = -0.508 \cdot$ VPD -0.276; $r^2 = 0.46$ *** ($p < 0.001$); RW-nI: $\Psi_l = -0.648 \cdot$ VPD -0.273; $r^2 = 0.92$ *** ($p < 0.001$). Regression lines for mandarin were: TW-fI: $\Psi_l = -0.466 \cdot$ VPD -0.132; $r^2 = 0.41$ *** ($p < 0.001$); TW-nI: $\Psi_l = -0.801 \cdot$ VPD -1.540; $r^2 = 0.84$ *** ($p < 0.001$); RW-fI: $\Psi_l = -0.532 \cdot$ VPD -0.172; $r^2 = 0.58$ *** ($p < 0.001$); RW-nI: $\Psi_l = -0.589 \cdot$ VPD -0.630; $r^2 = 0.70$ *** ($p < 0.001$).

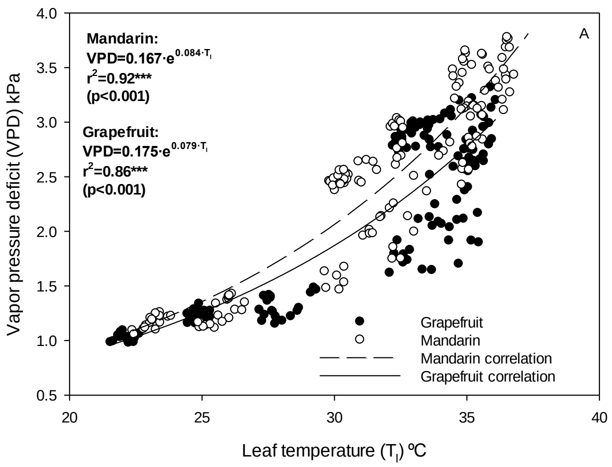

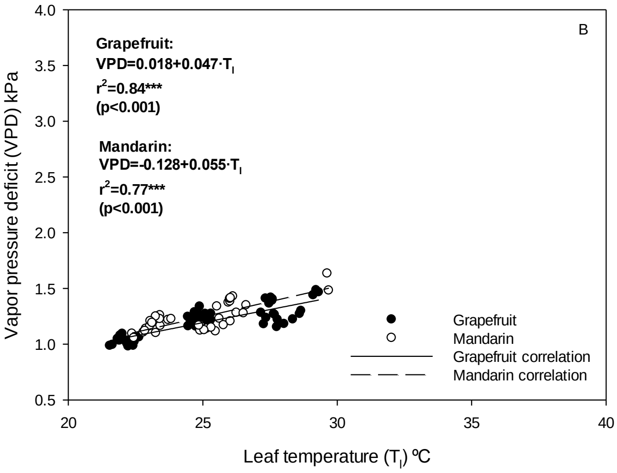

Figure S3. Relationship between vapor pressure deficit (VPD) and leaf temperature (T_l) for each crop (grapefruit and mandarin), with the exponential growth correlation for all data (**A**) and linear regression with data below 30 °C (**B**).

Table S1. Chemical parameters of each irrigation water source: Transfer Water (TW) and saline Reclaimed Water (RW). Each value is the mean of 12 individual measurements.

Parameter	Water source	
	TW	RW
pH	8.27±0.17	7.83±0.21
EC (dS·m^{-1})	0.93±0.14	3.73±0.80
NO_3^- (mg·L^{-1})	6.53±5.90	21.70±11.46
PO_3^{-4} (mg·L^{-1})	<1.0	4.05±2.17
K^+ (mg·L^{-1})	3.89±0.71	46.52±3.45
Ca^{+2} (mg·L^{-1})	93.67±16.73	166.59±14.07

Mg^{+} (mg·L^{-1})	40.95±7.09	122.21±22.10
B^{+3} (mg·L^{-1})	0.07±0.01	0.71±0.10
Na^{+} (mg·L^{-1})	46.03±14.18	604.03±121.65
Cl^{-} (mg·L^{-1})	69.95±22.04	639.31±189.91
SO_4^{-2} (mg·L^{-1})	195.76±53.61	604.46±159.42
